# Pregnancy Outcomes With and Without Adenomyomectomy in Infertile Patients With Adenomyosis: A Single‐Center Retrospective Study

**DOI:** 10.1111/jog.70149

**Published:** 2025-11-30

**Authors:** Fumika Hamaguchi, Yoshimi Kitawaki, Tsutomu Ohara, Taito Miyamoto, Asuka Okunomiya, Masumi Sunada, Yukiko Okada, Masaki Mandai, Akihito Horie

**Affiliations:** ^1^ Department of Gynecology and Obstetrics Hyogo Prefectural Amagasaki General Medical Center Amagasaki Hyogo Japan; ^2^ Department of Gynecology and Obstetrics Kyoto University Graduate School of Medicine Kyoto Japan; ^3^ Department of Gynecology and Obstetrics, Medical Research Institute Kitano Hospital Osaka Japan

**Keywords:** adenomyomectomy, adenomyosis, double flap, infertility, triple flap

## Abstract

**Aim:**

To evaluate pregnancy outcomes by treatment approach and assess the appropriateness and safety of surgical intervention in patients with infertility and uterine adenomyosis.

**Methods:**

A retrospective analysis was conducted on patients, diagnosed with adenomyosis at our institution from 2013 to 2023, who desired conception. The study population was divided into two groups by the presence or absence of surgical intervention, and the type of adenomyosis lesions, infertility treatment modalities, and pregnancy outcomes were evaluated. For patients who underwent adenomyomectomy, operative methods, pre‐ and postoperative endometrial thickness and dysmenorrhea severity, and perinatal complications in postsurgical pregnancies, were assessed.

**Results:**

Twenty‐one patients with adenomyosis (10 non‐surgical; 11 surgical) were analyzed. All patients in the non‐surgical group had focal adenomyosis lesions, and 70% achieved pregnancy through intrauterine insemination or assisted reproductive technology, with a 50% live birth rate. Most surgical patients had diffuse adenomyosis lesions and endometrial thinning in the luteal phase. Seven patients underwent triple‐flap surgery, while four underwent double‐flap surgery; 36% of surgical patients achieved pregnancy through assisted reproductive technology, with an 18% live birth rate. All patients showed improvement in postoperative dysmenorrhea and a significant increase in luteal phase endometrial thickness. No uterine rupture occurred in postsurgical pregnancies, but one patient had a late miscarriage and placenta accreta.

**Conclusions:**

Aggressive management of severe uterine adenomyosis with diffuse lesions through adenomyomectomy, followed by assisted reproductive technology, may be effective. Adenomyomectomy potentially enhances fertility by improving dysmenorrhea and thin endometria. However, careful management of postoperative pregnancies is necessary, considering perinatal complications.

## 1 Introduction

Adenomyosis is a benign uterine disorder, characterized by diffuse invasion and proliferation of endometrial tissue within the myometrium and accompanied by dysmenorrhea and hypermenorrhea [1]. It frequently coexists with endometriosis [2]. Deformation of the uterine cavity and the disruption of the normal junctional zone structure by adenomyosis lead to excessive uterine peristalsis, which affects sperm and embryo transport. Adenomyotic lesions cause abnormal endometrial function, which affects embryo implantation; thus, adenomyosis is considered to cause infertility [3, 4, 5]. A meta‐analysis of assisted reproductive technology (ART) outcomes in patients with infertility and adenomyosis found that patients with adenomyosis had a 28% lower pregnancy rate and a 2.12‐fold increased risk of miscarriage than did those without adenomyosis [6]. Furthermore, the miscarriage rate has been reported to be higher in patients with adenomyosis in embryo transfer of donated oocytes [7]. These findings suggest that adenomyosis may contribute to infertility by affecting oocyte quality and that the risk of miscarriage due to uterine factors is also significant. Therefore, therapeutic intervention for adenomyotic lesions is recommended for patients with adenomyosis who desire to conceive, to mitigate infertility and post‐conception complications, such as miscarriage. However, hormonal therapies are often associated with prolonged treatment times for lesion reduction, risk of early regrowth of lesions and symptom flare‐up after discontinuation, and consequent interruptions of fertility treatments. Fertility‐preserving surgery is therefore considered an alternative approach to avoid these issues.

Fertility‐sparing surgery for adenomyosis includes excision of the adenomyotic lesion and enucleation of adenomyosis (adenomyomectomy) to reduce the lesion. The following techniques have been developed and utilized: wedge resection for focal lesions [8, 9]; reduction of diffuse lesions [10]; and triple‐flap [11] and double‐flap methods and their variations [12, 13] for diffuse lesions. Although the effect of adenomyomectomy on infertility has not been conclusively demonstrated, high pregnancy rates have been reported after adenomyomectomy, partly due to the contribution of ART in postoperative treatment [14]. However, postoperative complications of adenomyomectomy include uterine rupture and placenta accreta, posing a safety issue [14, 15].

The triple‐flap technique is an adenomyomectomy procedure developed by Osada et al. [11] with the intention of reconstructing the uterine myometrium for postoperative pregnancy. This procedure is characterized by (1) the use of a cold knife and minimal use of energy devices to prevent delayed wound healing due to thermal damage to the tissue surrounding the lesion, and by (2) avoidance of overlapping suture lines to construct a uterine wall of appropriate thickness after thorough removal of the lesion. This method is expected to reduce the risk of uterine ruptures.

The triple‐flap technique is advantageous for wound repair after lesion removal. In cases in which the triple‐flap method is not suitable or feasible because of the size of the lesion, the double‐flap technique can be used, in which the suture lines are shifted in a similar manner, using a cold knife as in Osada's method. In addition, due to the background described above, infertility can be treated without surgery in patients with relatively mild lesions that are deemed to have minimal impact on embryo implantation and pregnancy outcomes, while adenomyomectomy can be performed in patients with extensive adenomyosis and endometrial thinning. However, clear treatment guidelines for infertility and adenomyosis are lacking, and which patients should be considered candidates for surgery remains unclear. Moreover, reports on the pregnancy outcomes following adenomyomectomy are limited, especially for the triple‐flap procedure or similar techniques.

Therefore, in this study, we aimed to evaluate the appropriateness of the surgical indication for adenomyomectomy and the efficacy and safety of the triple‐ and double‐flap techniques by analyzing the choice of surgical intervention and pregnancy outcomes in patients with adenomyosis who desired to conceive, as well as perinatal complications in pregnancies after adenomyomectomy.

## 2 Methods

### 2.1 Study Population and Data Collection

A retrospective study of patients diagnosed with uterine adenomyosis at Kyoto University Hospital from January 2013 to December 2023 was conducted. During this period, adenomyomectomy was indicated for cases in which adenomyotic lesions were extensive and accompanied by luteal‐phase endometrial thinning, as well as in cases without such thinning but in which adenomyosis was clearly contributing to infertility or miscarriage and was difficult to manage with hormonal therapy. The study was approved by the Ethics Committee of Kyoto University Hospital (approval number: R4081). The study was disclosed to the patients, allowing them to opt out; none declined participation. Patients with uterine adenomyosis who desired pregnancy and had undergone infertility treatment or follow‐up for pregnancy were included in the study to evaluate the therapeutic efficacy of adenomyomectomy and pregnancy outcomes thereafter. The following information was collected from the patients' surgical and medical records: age at initiation of treatment, localization and size of adenomyosis lesions, presence or absence of surgical treatment, reason for surgical treatment, surgical methods, pre‐ and postoperative endometrial thickness and degree of dysmenorrhea, history of infertility treatment, method of conception, and pregnancy outcome. The data of patients who had undergone adenomyomectomy but no longer wished to conceive, and those of patients who had a history of surgical treatment for adenomyosis by a previous physician, were excluded.

The localization of adenomyosis lesions was evaluated by MRI and ultrasound imaging; lesions were defined as focal if more than 25% of the normal muscle layer was present around the lesion, and diffuse if it was not [16]. Clinical pregnancy was defined as the identification of the gestational sac in the uterus at 6 weeks. To examine changes in dysmenorrhea symptoms with surgery, the degree of menstrual pain was quantified using the Numerical Rating Scale (NRS), an 11‐point scale ranging from 0 to 10, with 0 representing no pain and 10 representing the worst possible pain. Endometrial thickness was measured using transvaginal ultrasound in the luteal phase of the menstrual cycle before and after surgery; in premedicated cases, the first measurement was done before gonadotropin‐releasing hormone analog (GnRH‐a) initiation.

### 2.2 Statistical Analysis

Pre‐ and postoperative NRS scores and endometrial thicknesses were compared using the corresponding *t*‐tests. Statistical analysis was performed using Prism 9 (GraphPad Software, Boston, MA, USA), with the exclusion of one and two cases in which no pre‐ or postoperative data were available for the NRS and endometrial thickness, respectively.

## 3 Results

### 3.1 Patient Backgrounds, Treatments, and Pregnancy Outcomes

**FIGURE 1:**
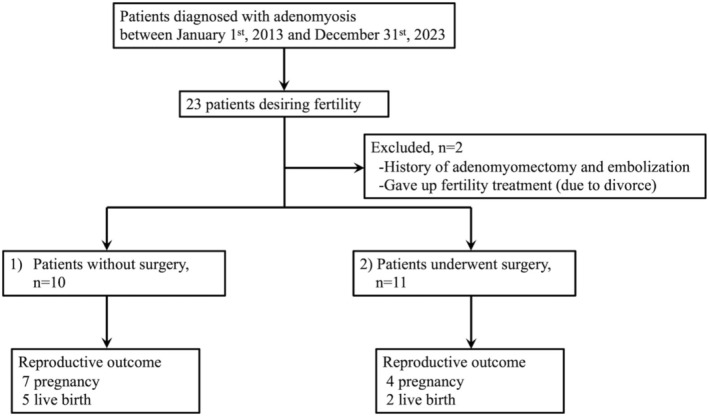
Study flow chart. Flowchart showing participants and exclusions in this study. After exclusions, 21 cases were included in the analysis.

Of the patients diagnosed with adenomyosis, 21 expressed a desire for conception, had no previous surgical intervention, and had undergone pregnancy follow‐up or infertility treatment. Ten (48%) patients had not undergone surgery and 11 (52%) had undergone surgery (Figure 1).

All the patients who had not undergone surgery had focal adenomyosis lesions, and the mean age at the start of treatment was 36.8 years; five (50%) were nulliparous (Table 1). Of the 10 patients who underwent surgery, two were treated with intrauterine insemination (IUI), eight were treated with ART, and four underwent hormonal therapy with GnRH agonists or antagonists during infertility treatment for worsening menstrual pain or increased lesions. Seven (70%) patients became pregnant; two (20%) had early miscarriages, and five (50%) resulted in live births. Two pregnancies were achieved through IUI and five through ART, with a mean time to conception of 19.7 months (Table 2).

**TABLE 1:** Characteristics of patients who did not undergo surgery (*n* = 10) and patients who underwent surgery (*n* = 11).

Characteristics	Non‐surgical group	Surgical group
Age (years)	36.8 (32–40)	38.9 (35–42)
Gravidity	5/10 (50.0%)	5/11 (45.5%)
Parity	5/10 (50.0%)	0/11 (0.0%)
Miscarriage	2/10 (20.0%)	4/11 (36.4%)
Type of adenomyosis		
Focal (posterior wall)	10/10 (100.0%)	1/11 (9.1%)
Maximum size of adenomyoma (mm)	40.4 (30–58)	47.0
Diffuse	0/10 (0.0%)	10/11 (90.9%)
Unilateral wall	—	8/11 (72.7%)
Bilateral walls	—	2/11 (18.2%)
Endometrial thickness (mm)	12.4 (9.0–14.8)	7.1 (4.1–14.1)
Preoperative infertility time (months)	—	19.1 (2–36)
Preoperative infertility treatment		
None	—	4/11 (36.4%)
IUI	—	1/11 (9.1%)
ART	—	6/11 (54.5%)
Reason for surgery		
Thin endometrium	—	7/11 (63.6%)
Others^a^	—	4/11 (36.4%)

*Note:* Data are presented as the mean (minimum–maximum) or *n* (%).

^a^ Severe dysmenorrhea, repeated ART failure (without thin endometrium), history of miscarriage due to adenomyosis infection, repeated miscarriage.

**TABLE 2:** Fertility outcomes of patients who did not undergo surgery (*n* = 10) and postoperative fertility outcomes of patients who underwent surgery (*n* = 11).

Outcomes	Non‐surgical group	Surgical group
Surgical methods		
Triple flap	—	7/11 (63.6%)
Double flap	—	4/11 (36.4%)
Follow‐up time (months)	24.1 (1–64)	—
Postoperative follow‐up time (months)	—	17.7 (1–53)
Infertility treatment^a^		
TI	0/10 (0%)	1/11 (9.1%)
IUI	3/10 (30.0%)	1/11 (9.1%)
ART	7/10 (70.0%)	9/11 (81.8%)
Combined hormonal treatment (GnRH‐a)	4/10 (40.0%)	3/11 (27.3%)
Pregnancy	7/10 (70.0%)	4/11 (36.4%)
Live birth	5/10 (50.0%)	2/11 (18.2%)
Time to pregnancy (months)	19.7 (1–50)	7.3 (1–16)
Method of conception		
IUI	2/7 (28.6%)	0/4 (0%)
ART	5/7 (71.4%)	4/4 (100%)

*Note:* Data are presented as the mean (minimum–maximum) or *n* (%).

Abbreviations: ART, assisted reproductive technology; GnRH‐a, gonadotropin‐releasing hormone analog; IUI, intrauterine insemination; TI, timed intercourse.

^a^ Infertility treatment: non‐surgical group, during follow‐up; surgical group, postoperatively.

Of the 11 patients who underwent surgical treatment, 10 had diffuse adenomyosis lesions; in two, the entire uterus was involved, and eight had diffuse lesions of either the anterior or posterior uterine walls. The mean age at the start of treatment was 38.9 years, and all the patients were nulliparous. Seven (64%) patients received infertility treatment prior to surgery, six involving ART. Most exhibited endometrial thinning, with preoperative luteal endometria < 8 mm. Other indications for surgery were severe dysmenorrhea, repeated failure of embryo transfer (without endometrial thinning), a history of miscarriage due to adenomyosis infection, and recurrent miscarriage in one case each (Table 1).

Seven patients underwent adenomyomectomy with the triple‐flap method, and the remaining four underwent adenomyomectomy with the double‐flap method. Postoperative infertility treatment comprised timed intercourse in one case, IUI in one case, and ART in nine cases, resulting in four pregnancies (36%) and two live births (18%). The mean time to conception was 7.3 months, and all four pregnancies were achieved by ART. Forty‐four percent of the patients who underwent ART after adenomyomectomy conceived (Table 2). Cases in which pregnancy was not achieved following postoperative infertility treatment were those with diminished ovarian reserve due to complications of endometriosis or advanced maternal age (40 years or older). Among the seven patients who did not conceive postoperatively, five (71%) had coexisting endometriosis, a prevalence higher than that among the four patients who did achieve pregnancy postoperatively (25%).

### 3.2 Effect of Surgery on Endometrial Thickening and Menstrual Pain

**FIGURE 2:**
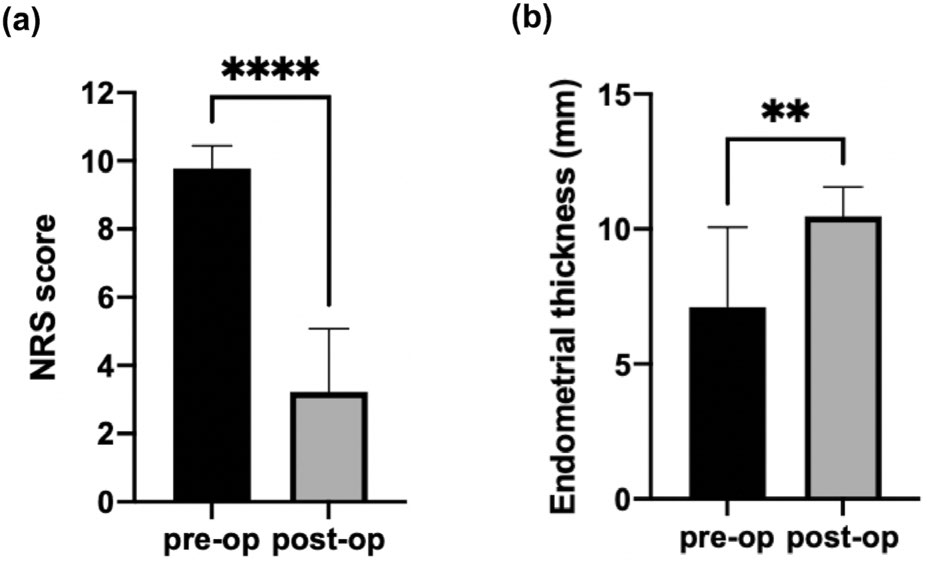
Preoperative and postoperative dysmenorrhea (a) and endometrial thickness in the luteal phase (b) in the surgery group. Data for each group (*n* = 9) are presented. The graphs show mean ± standard deviation. Pre‐op, preoperative; post‐op, postoperative. **p < 0.01, ****p < 0.0001.

**FIGURE 3:**
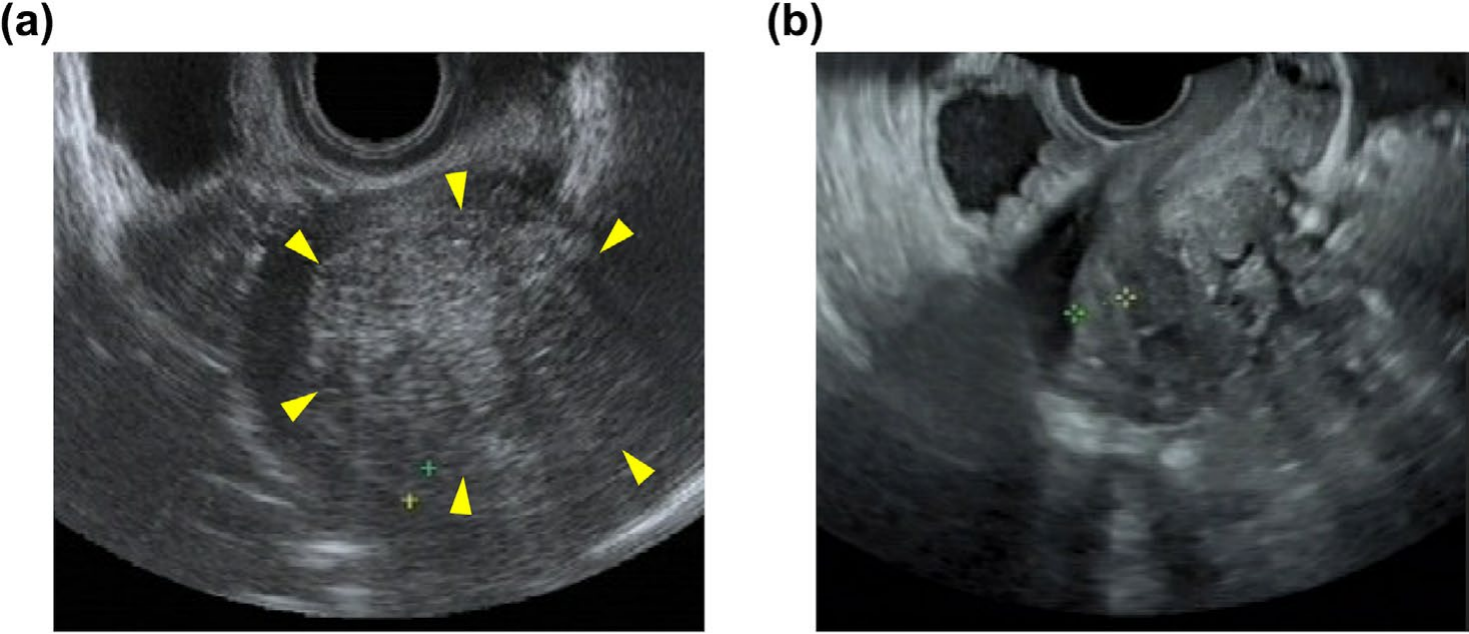
Changes in uterine morphology and endometrial thickness post‐adenomyomectomy. Representative images of the pre‐ and post‐adenomyomectomy uterus in luteal phases are presented; (a) preoperative; (b), postoperative. The yellow arrows indicate the adenomyosis lesion. The area between the two plus marks (+) indicates the endometrium.

The postoperative NRS scores improved in all the patients who underwent surgery, compared with the preoperative NRS scores (*p* < 0.0001) (Figure 2a). Postoperative luteal phase endometrial thickness also showed a significant increase (*p* < 0.01) (Figures 2b, 3). Eight of the nine patients (89%) for whom preoperative endometrial thickness data were available had thicker endometria in the postoperative luteal phase than those in the preoperative cycle, especially in patients with a preoperative endometrial thickness < 8 mm.

### 3.3 Perinatal Complications of Postoperative Pregnancy

Two of the surgical cases that resulted in live births, delivery was via cesarean section, with no uterine rupture or placenta accreta. The mean gestational age at delivery was 36 weeks. Both patients were treated for threatened preterm labor, and one patient underwent an emergency cesarean section at 35 weeks due to increased uterine contractions. Of the two pregnancies that did not result in live births, one was a late miscarriage at 15 weeks of gestation, likely due to insufficient myometrial distension. An adherent placenta was identified at delivery, necessitating manual removal of the placenta and subsequent bilateral uterine artery embolization due to severe postpartum hemorrhage (Table 3). Postoperative MRI in this case revealed uterine cavity deformation (Figure S1). The other case involved a patient with advanced age who had an early miscarriage, at 8 weeks' gestation.

**TABLE 3:** Perinatal outcomes in patients who underwent surgery.

Age (years)	Type of adenomyosis	Surgical methods	Complications	Treatments	Delivery method	Gestation at delivery (weeks)	Birth weight (g)
35	Diffuse (anterior wall‐fundus)	Double flap	Spontaneous abortion at 15 weeks placenta accreta	Interventional radiology	—	—	—
39	Diffuse (posterior wall)	Triple flap	Preterm labor	Tocolytic treatment	Emergency CS	35	2306
40	Diffuse (bilateral wall)	Triple flap	Threatened preterm labor	Tocolytic treatment	Elective CS	37	3426

Abbreviation: CS, cesarean section.

## 4 Discussion

In this retrospective study conducted at a single institution, we found that adenomyomectomy using triple‐ or double‐flap methods significantly reduced menstrual pain, resulted in adequate postoperative thickening of the luteal phase endometrium in all patients with thin endometria, and yielded a high pregnancy rate with postoperative ART. This is the first study to demonstrate mitigation of endometrial thinning following adenomyomectomy. Additionally, patients with focal adenomyosis and no endometrial thinning exhibited favorable fertility outcomes without surgical intervention.

The reported pregnancy rates following adenomyomectomy range from 17.5% to 72.7%, with most reported pregnancies achieved through ART [14]. The results of this study are consistent with those of previous reports and suggest that adenomyomectomy may be an efficacious treatment for patients with severe adenomyosis who desire to conceive.

In cases of diffuse adenomyosis, or focal adenomyosis with extensive lesions, sufficient endometrial thickness for embryo implantation is frequently not achieved. It is well established that endometrial thickness is associated with fertility outcomes, and an endometrial thickness of less than 8 mm results in a reduced pregnancy rate with ART [17]. In this study, 7 of the 11 patients who underwent surgery exhibited preoperative luteal phase endometria less than 8 mm, and although some had endometria less than 6 mm, rendering pregnancy improbable, sufficient endometrial thickness in the luteal phase was achieved after surgery in all patients with thin endometria. In cases of severe adenomyosis, endometrial thinning is frequently observed, presumably due to the inability of the endometrium to thicken because of pressure from the dense adenomyotic tissue. Although the relationship between alterations in endometrial thickness due to adenomyosis and infertility remains unclear, removal of the adenomyotic lesions might have ameliorated endometrial thinning and thereby contributed to improved fertility outcomes. Notably, a high proportion of patients who did not achieve pregnancy postoperatively had endometriosis, indicating that non‐uterine factors, such as diminished ovarian reserve, likely influenced postoperative reproductive outcomes.

Conversely, half of the patients who did not undergo surgery, all of whom had focal adenomyosis, achieved live births. Patients with focal adenomyosis with manageable menstrual pain and whose uterine lumen is not severely deformed can achieve pregnancy without surgical intervention and can be safely managed during the perinatal period. Regarding the localization of adenomyotic lesions, diffuse lesions have been reported to increase perinatal complications such as second‐trimester miscarriage, preterm premature rupture of membranes, and preeclampsia [16], while focal lesions are associated with primary infertility [18]; both types negatively affect fertility and pregnancy outcomes. Although no definitive indications for adenomyomectomy exist, we have considered surgical intervention for patients with long‐term failure of ART, difficulty in pain control, and luteal phase endometrial thickness less than 8 mm, while considering the complications of adenomyomectomy. Conversely, patients with relatively mild, focal adenomyosis were primarily treated for infertility without surgery. Based on the pregnancy outcomes of patients who did not undergo surgery in this study, these criteria for surgery seem appropriate.

One of the disadvantages of adenomyomectomy is the increased rate of uterine rupture and placenta accreta in postoperative pregnancies, which present a significant challenge in perinatal management. The incidence of uterine rupture in pregnancies following adenomyomectomy is reported to be 3.6% [14]; this incidence is higher than the 0.27%–0.7% reported for a trial of labor after cesarean section [19] and the 0.7%–1.0% reported for pregnancies following myomectomy [20]. The extent of uterine adenomyosis removal, that is, adenomyosis remnants in the tissue, the extent and size of the myometrial defect, and the method of uterine cavity and uterine wall reconstruction, are associated with uterine rupture post‐adenomyomectomy [14]. The reported risk of placenta accreta in pregnancies following adenomyomectomy is 6.2%–9.1% [15, 21]. Given that advanced adenomyosis often requires treatment with ART, and given that the reported rate of placenta accreta in ART pregnancies is 2%–3% [22, 23, 24], the risk of placenta accreta post‐adenomyomectomy is high. Since uterine rupture and placenta accreta are life‐threatening complications for both mother and fetus, the indication for adenomyomectomy should be carefully considered.

The optimal duration of contraception post‐adenomyomectomy has not been established. In the postoperative wound healing process after myomectomy, alterations in uterine blood flow occur concomitantly with angiogenesis on the seventh postoperative day. The myometrium and endometrium morphology and wound blood flow return to normal 3 months after surgery if no large hematoma is formed [25, 26, 27]. Furthermore, by selecting sutures that can be absorbed within 90 days, there will be no foreign body residue at 3 months post‐surgery, rendering this the earliest opportunity to consider pregnancy. Thus, at our institution, hysteroscopy and contrast‐enhanced MRI are performed 4–6 months post‐surgery. If these confirm a normal endometrial appearance, an absence of adhesions in the uterine lumen, and normal blood flow of the myometrium at the surgical site, pregnancy is permitted.

In this study, one of the pregnancies exhibited an adherent placenta. In this case, although the postoperative examination confirmed restoration of myometrial blood flow and a normal endometrium, luminal deformation was observed. In adenomyomectomy, particularly in cases where myometrial repair is difficult or where postoperative myometrial deformity is observed, attention should be paid to the potential for post‐pregnancy complications.

A limitation of this study is that, as our institution prioritizes surgical treatment as the first‐line option for severe adenomyosis, a comparison with pregnancy outcomes following hormonal therapies in similar patients could not be performed. Additionally, the surgical and non‐surgical groups varied substantially in clinical backgrounds, such as age and the severity of adenomyosis, which precludes statistical analysis regarding the impact of surgical intervention. To rigorously evaluate the efficacy of adenomyomectomy, a prospective study comparing surgical treatment with GnRH‐a therapy in cases of severe adenomyosis is warranted. Furthermore, the safety of pregnancy, particularly the incidence of postoperative uterine rupture, remains insufficiently investigated due to the limited number of cases in this study. A large‐scale multicenter study is warranted to evaluate the efficacy of the triple‐flap method in preventing uterine rupture.

In conclusion, the triple‐ or double‐flap technique for adenomyomectomy may not only reduce dysmenorrhea, and consequently improve fertility, but may also enhance fertility by mitigating endometrial thinning. For severe adenomyosis with diffuse lesions, aggressive treatment with adenomyomectomy and subsequent ART is advisable. However, because of the risk of complications associated with surgery, careful selection of patients for adenomyomectomy is essential, and treatment strategies should be tailored to each individual case.

## Author Contributions

**Fumika Hamaguchi:** data curation, investigation, visualization, writing – original draft, writing – review and editing, formal analysis. **Yoshimi Kitawaki:** data curation, investigation, visualization, writing – review and editing, writing – original draft, formal analysis. **Tsutomu Ohara:** conceptualization, writing – review and editing. **Taito Miyamoto:** methodology, writing – review and editing. **Asuka Okunomiya:** conceptualization, writing – review and editing. **Masumi Sunada:** conceptualization, writing – review and editing. **Yukiko Okada:** methodology, writing – review and editing. **Masaki Mandai:** conceptualization, supervision, writing – review and editing. **Akihito Horie:** conceptualization, methodology, data curation, writing – original draft, writing – review and editing, supervision.

## Funding

The authors have nothing to report.

## Ethics Statement

The study was approved by the Ethics Committee of Kyoto University Hospital (approval number: R4081).

## Consent

The study was disclosed to the patients, allowing them to opt out. No written consent has been obtained from the patients as this was a retrospective study using anonymized medical records.

## Conflicts of Interest

The authors declare no conflicts of interest.

## Supporting information


**Figure S1:** Pre‐ and postoperative pelvic MRI of a case with postoperative placenta accreta. Preoperative (A) and postoperative (B) T2‐weighted images are presented. Left, axial sections; right, sagittal sections.

## Data Availability

The data that support the findings of this study are not publicly available, as consent for data sharing in a public repository was not obtained from the study participants and uploading the data would compromise ethical standards. Additional data related to this study are available from the corresponding author upon reasonable request.
