# Supplementary figures and images for: Pregnancy Outcomes With and Without Adenomyomectomy in Infertile Patients With Adenomyosis: A Single‐Center Retrospective Study
Source: J Obstet Gynaecol Res. 2025 Nov 30;51(12):e70149. doi: 10.1111/jog.70149 (PMC12665454; doi:10.1111/jog.70149)

**(a)**

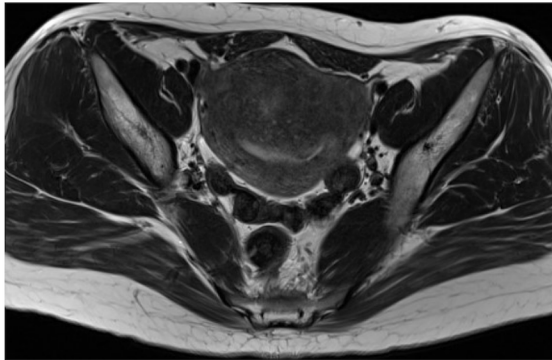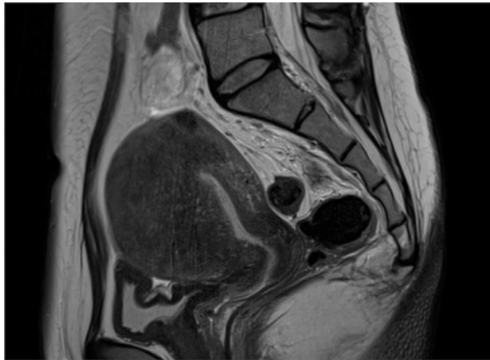

**(b)**

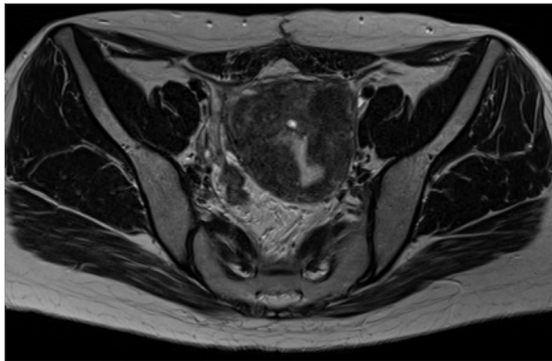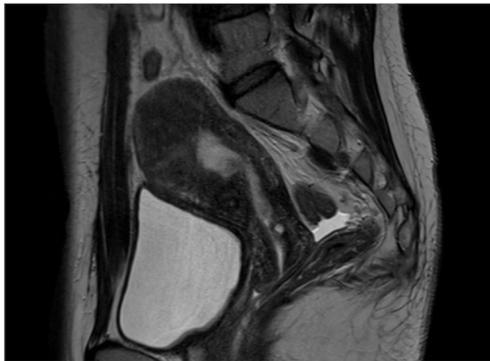

Supplement: Supplementary file 1 — Figure S1: Pre‐ and postoperative pelvic MRI of a case with postoperative placenta accreta. Preoperative (A) and postoperative (B) T2‐weighted images are presented. Left, axial sections; right, sagittal sections. [file JOG-51-0-s001.pdf]
